# Acquisition of High Spectral Resolution Diffuse Reflectance Image Cubes (350–2500 nm) from Archaeological Wall Paintings and Other Immovable Heritage Using a Field-Deployable Spatial Scanning Reflectance Spectrometry Hyperspectral System

**DOI:** 10.3390/s22051915

**Published:** 2022-03-01

**Authors:** Roxanne Radpour, John K. Delaney, Ioanna Kakoulli

**Affiliations:** 1 Scientific Research Department, National Gallery of Art, Washington, DC 20565, USA; 2 Materials Science and Engineering, University of California, Los Angeles, CA 90095, USA; kakoulli@ucla.edu

**Keywords:** hyperspectral imaging, reflectance spectroscopy, imaging spectrometry, archaeometry, field remote sensing, monumental wall painting, pigment analysis, chemical mapping

## Abstract

There is growing interest in bringing non-invasive laboratory-based analytical imaging tools to field sites to study wall paintings in order to collect molecular information on the macroscale. Analytical imaging tools, such as reflectance imaging spectrometry, have provided a wealth of information about artist materials and working methods, as well as painting conditions. Currently, scientific analyses of wall paintings have been limited to point-measurement techniques such as reflectance spectroscopy (near-ultraviolet, visible, near-infrared, and mid-infrared), X-ray fluorescence, and Raman spectroscopy. Macroscale data collection methods have been limited to multispectral imaging in reflectance and luminescence modes, which lacks sufficient spectral bands to allow for the mapping and identification of artist materials of interest. The development of laboratory-based reflectance and elemental imaging spectrometers and scanning systems has sparked interest in developing truly portable versions, which can be brought to field sites to study wall paintings where there is insufficient space or electrical power for laboratory instruments. This paper presents the design and testing of a simple hyperspectral system consisting of a 2D spatial spot scanning spectrometer, which provides high spectral resolution diffuse reflectance spectra from 350 to 2500 nm with high signal to noise and moderate spatial resolution (few mm). This spectral range at high spectral resolution was found to provide robust chemical specificity sufficient to identify and map many artists’ materials, as well as the byproducts of weathering and conservation coatings across the surface of ancient and Byzantine Cypriot wall paintings. Here, we present a detailed description of the hyperspectral system, its performance, and examples of its use to study wall paintings from Roman tombs in Cyprus. The spectral/spatial image processing workflow to make maps of pigments and constituent painting materials is also discussed. This type of configurable hyperspectral system and the imaging processing workflow offer a new tool for the field study of wall paintings and other immovable heritage.

## 1. Introduction

For on-site scientific analyses of archaeological wall paintings and other painted monuments, there is an increased interest for reliable, non-destructive, and non-invasive technologies to analyze painting materials such as pigments and binding media, understand painting production technologies, and identify regions of degradation and products of alteration as well as conservation materials from previous treatments [1,2,3,4]. These polychrome surfaces are challenging to study, as they are integral parts of walls and structures and therefore are exposed to anthropogenic and environmentally-linked events. Due to ongoing deterioration of the paint layers from aging, weathering, and biological effects, the decorated surfaces are left in a fragile state and suffer major paint loss, resulting in having trace amounts of colorants remaining or becoming obscured [5,6,7,8,9]. On-site analyses are further restricted by challenging physical spaces for large-sized equipment and have time constraints for the setup and study of the paintings, as they are often located in highly-visited tourist sites or serve religious purposes. To overcome these challenges, portable hand-held, point-based characterization techniques provide an effective solution for fast and reliable non-invasive field investigations of archaeological paintings. These include: reflectance spectroscopy in the near-ultraviolet (near-UV, here 300 to 400 nm), visible (400 to 750 nm), near-infrared (NIR, 750 to 1000 nm), and mid-IR (2.5 to 20 mm); X-ray fluorescence (XRF) spectroscopy; Raman spectroscopy; and Fourier-transform infrared spectroscopy (FTIR) [10,11,12]. Combinations of these techniques are often employed in tandem to provide complementary data for painting materials assignment [13,14,15,16,17,18]. While the information provided by these techniques is important, a comprehensive understanding of the high spatial variability of materials and complex stratigraphy of ancient wall paintings consisting of a single layer or multiple layers of paint composed of one or more colorants/pigments and the binding medium/media is restricted, as most robust techniques use spot analysis. Information on the extent of decoration and materials distribution is lacking through these approaches. Techniques capable of providing spatially-resolved data such as forensic photography (near-UV-induced and visible-induced luminescence, color, and NIR) using modified commercial color cameras or multiband imaging provide no or limited specific chemical data, though they can be used to visualize the distribution of some pigments. Diffuse reflectance multispectral imaging (MSI, tens of bands) [19,20] lacks sufficient spectral resolution and spectral bands to accurately identify and map artist’s materials, but can often be used to spatially separate many pigments.

Reflectance imaging spectrometry systems or reflectance hyperspectral imaging systems, on the other hand, produce a high-resolution spectrum for each spatial pixel of a captured image scene [21,22] and offer an attractive solution to these analytical limitations. These systems produce 3D image cubes (*x*,*y*—spatial dimensions, *z*—spectral dimension), which can be data-mined through multivariate statistical analysis to identify a set of unique spectral signatures, or endmembers, which can then be mapped across the image scene to show how these endmembers are spatially distributed [23]. The application of reflectance imaging spectrometers, utilizing principles of diffuse reflectance spectroscopy from the visible to the NIR, or further into short-wave infrared (SWIR, 1000 to 2500 nm) [24,25,26] to study artifacts of cultural and artistic value has provided important insight into historical paintings and manuscripts, including materials identification, regions of degradation, painting alterations, and prior compositions [27,28,29,30,31]. For example, access to the near-UV is useful to identify different white pigments such as titanium oxide (titanium white, TiO_2_) and zinc oxide (zinc white, ZnO_2_), which can be found in wall paintings as overpainting or restoration, by using the location of the inflection points of their respective band gap transitions [32]. Anatase and rutile, both naturally occurring forms of TiO_2_, have different band gap energy transitions with approximately a 20–30 nm separation [33,34], which can be distinguished using reflectance spectrometry systems with sufficiently high spectral resolution. Diffuse reflectance spectroscopy from the near-UV through the visible into the NIR also provides pigment-specific electronic transitions that can identify many inorganic and organic pigments found in wall paintings [35]. Operation in the SWIR spectral range enables the recording of overtones and combination bands associated with chemical functional groups, supporting the identification of pigments (i.e., hydroxyls, carbonates) [30], paint binders (lipidic bands, protein bands, carbohydrates) [36], degradation products and invasive species (e.g., salts and microorganisms, respectively) [7,37], and organic surface conservation materials (Paraloid-B72, waxes, gels, etc.) [38,39]. This allows visualizations of the distribution of the original artist materials, degradation products, and conservation materials from previous restoration interventions, and improves detection of organic and inorganic pigments, clays, and organic binding media [40,41,42,43,44,45]. Azurite (Cu_3_(CO_3_)_2_(OH)_2_), a mineral found extensively in historical wall paintings, manuscripts, and fine art paintings, is an important example of a pigment with diagnostic features in both the VNIR and SWIR spectral ranges that are useful for its characterization. Azurite has OH stretching and bending vibrational overtones at 1495, 2285, and 2352 nm [46,47] that are useful to help identify the pigment and possibly map its presence more accurately than with VNIR if it has been mixed with other pigments, layered, or chemically altered [48]. These applications emphasize the importance of bringing high-resolution reflectance imaging spectrometry capabilities spanning the near-UV to SWIR spectral range into the field for wall painting analyses.

**Figure 1:**
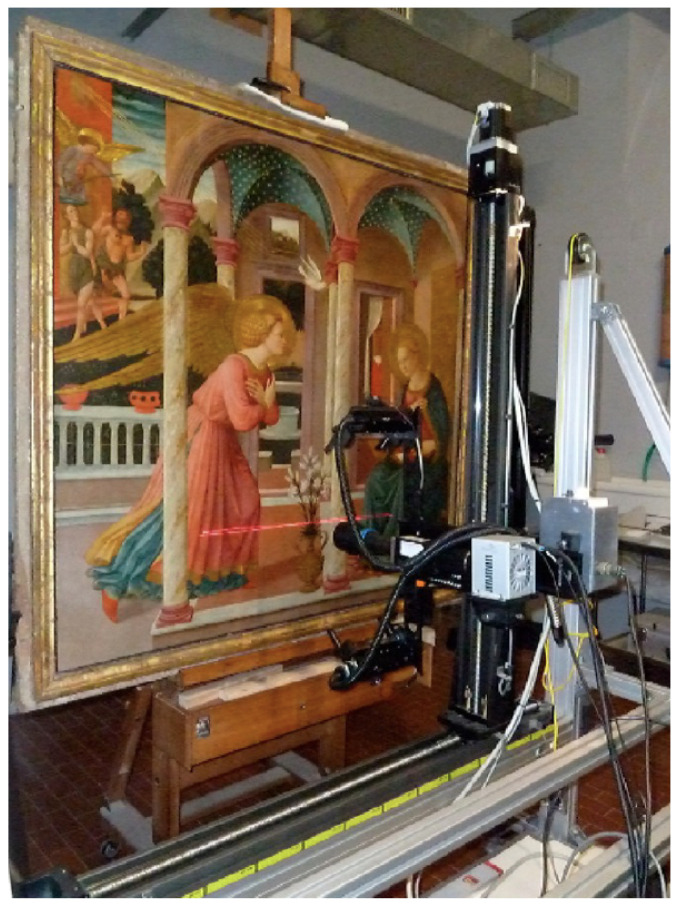
L’Istituto di Fisica Applicata “Nello Carrara” (IFAC-CNR), Florence, Italy, reflectance hyperspectral imaging system (400 to 1700 nm). Reprinted (adapted) with permission from [27]. Copyright 2016 American Chemical Society.

Currently, commercially-available and laboratory-developed reflectance hyperspectral systems for the study of meter-sized paintings are not well-suited for field deployment and are expensive if they operate from the visible into the SWIR. While many of the developed instruments are transportable, they are large and heavy, requiring special transportation and lengthy set-up time as well as a source of stable electrical power. Though such instruments (e.g., Figure 1) provide good spectral (<3 to 7 nm) and high spatial resolution (<0.25 mm), there are limitations in their ability to be applied in the field. Moreover, if the goal is to identify and map artist materials in wall paintings, particularly those that do not have fine decorative details similar to cases requiring remote sensing hyperspectral cameras, then there is a greater need for high spectral resolution and higher signal to noise at the expense of high spatial sampling [21].

**Figure 2:**
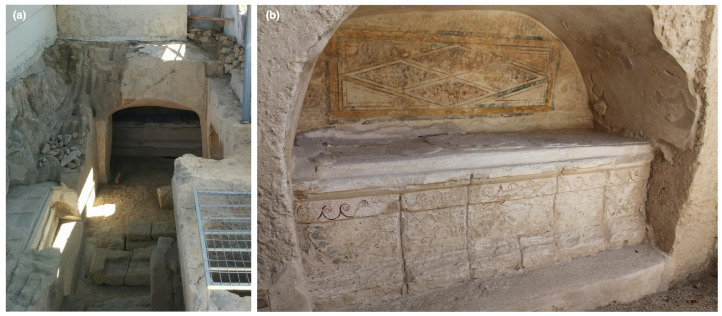
(**a**) Tomb 3882, uncovered and partially damaged during a modern-day construction project. The southeast arcosolium (**b**) located on the right, where a painting of interest for this study is located, is a confined space. The limited physical access to the tympanum, the care needed to operate around the surrounding decoration on the dado level, and the lack of electrical power create a significant engineering challenge for bringing in and configuring large/heavy instrumentation to scan the painting.

Many archaeological sites such as tombs do not offer ample space for the setup of complex instruments, or they would at least require longer-term planning and potential re-engineering of the system to accommodate the available space. Figure 2, featuring a case study site for this paper, demonstrates this challenge. Furthermore, not all sites offer stable electrical power, which presents yet another problem.

Still, several studies utilizing reflectance hyperspectral imaging systems have demonstrated the usefulness of reflectance imaging spectrometry for field investigations of wall paintings: for example, Alfeld et al. (2018) applied visible-to-near-infrared (VNIR, 400 to 1000 nm, 2.8 nm spectral sampling) reflectance mapping on funerary wall paintings in the Theban Necropolis in Egypt to recover imagery and identify pigments used in their decoration [49]. The benefit of including portions of the SWIR spectral range for on-site studies to assess the degree of flaking and to recover faded mural patterns using multivariate statistical analyses was demonstrated on Chinese mural paintings (882 to 1719 nm, approximately 3.26 nm sampling, 320 × 400 spatial pixels) [50]. Another example of the potential utility of reflectance imaging spectrometry for archaeological field analyses to collect hyperspectral data over the 400 to 2500 nm spectral range was presented in Cucci et al. [51]. This study explored the type of information a state-of-the-art aerospace hyperspectral system (SIGMA, Leonardo Company, East Sussex, UK) could obtain from two Pompeiian wall paintings. In this paper, the authors were able to demonstrate the utility of lower spatial resolution (few mm) with high spectral sampling in the VNIR (1.2 nm) and moderate in the SWIR (5.8 nm) spectral ranges to map various materials such as iron oxide/oxyhydroxides (e.g., hematite (Fe_2_O_3_) and goethite (FeO(OH))) pigments, as well as gypsum (CaSO_4_·2H_2_O). Moreover, they were able to use principal component analysis to recover faded inscriptions in the VNIR spectral range. Owing to the disparity in the spatial sampling between the two imaging spectrometers (1024 pixels VNIR versus 320 pixels SWIR) the full spectral range was not exploited in the analysis. Finally, the need to fully illuminate the wall painting for the data collection and the resulting limitation on the signal to noise in the image cubes can be seen in the comparison of the reflectance spectra obtained from the image cube versus those obtained from a point fiber optics reflectance spectrometer (ASD Fieldspec FR Pro spectroradiometer, Malvern Panalytical, Malvern, UK). Collectively, these results, while impressive, underscore the critical tradeoff between high spatial resolution and both signal to noise and spectral fidelity over a broad spectral range for reflectance imaging spectrometry in the field.

To address the need for imaging spectroscopy applications for materials characterization and chemical mapping of wall paintings that remain on site, a custom-made scanning reflectance spectrometry (SRS) hyperspectral system was designed that can produce image cubes with high spectral sampling (1 to 2 nm) over a wide spectral range (350 to 2500 nm) at moderate spatial resolution (few mm). The system is portable with a configurable scan area, and can be battery operated. The SRS hyperspectral system consists of a rugged fiber-optic reflectance spectrometer and a customized computer-controlled 2D mechanical *x*-*y* scanner mounted on a tripod.

The utility of the SRS hyperspectral system to collect useable spectral image cubes with data quality sufficient for the identification and mapping of artists’ materials is demonstrated here by results from the analyses of two archaeological Cypriot wall paintings dated to the Hellenistic and Roman periods. The datasets are from an extensive study of funerary wall paintings, analyzed during field campaigns in the years 2017–2019, found in subterranean Hellenistic and Roman tombs present in/near ancient Nea Paphos (“new” Paphos) [52,53,54,55], the administrative and economic capital of the island of Cyprus during the Hellenistic and Roman time periods [56]. The recovered mosaics from the residences of the wealthy citizens such as that of the Roman governor of Cyprus at the Villa of Theseus are remnants of the thriving ancient city [57]. The location of Cyprus at the intersection of important civilizations in Europe, Asia, and Africa and the rich natural resources of the island brought about multiple transitions of power, infusions of new traditions, and exchanges of knowledge, crafts, wealth, and people [58,59,60,61]. Cyprus, whose name derives from the word “copper”, the main economic metal on the island mined since antiquity, was also known for green earth (mineral celadonite, (K(Mg,Fe^2+^)(Fe^3+^,Al)[Si_4_O_10_](OH)_2_)) as well as umbers and ochres (iron oxides/oxyhydroxides) [62,63]. For this reason, the scientific analysis of the Hellenistic and Roman paintings in Cyprus, for which relatively few technical studies exist [56,64,65,66], can provide invaluable insight into the use of these local minerals in painting, pigment synthesis methods, and trade of the raw materials to and from the island.

**Figure 3:**
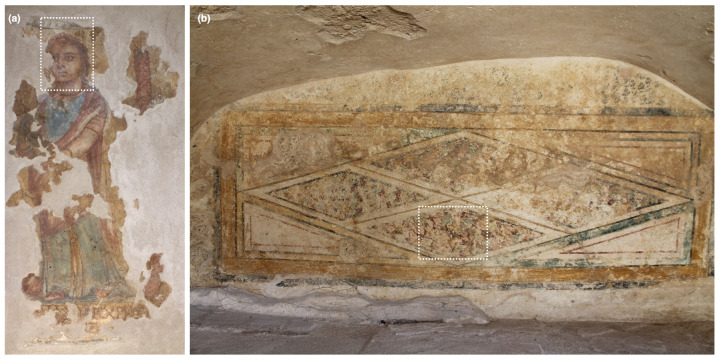
(**a**) *Painting of a woman*, from Tomb 3510 (Case Study 1). (**b**) Tympanum painting *Imitation marble* on the southeast arcosolium of Tomb 3882 (Case Study 2). The scanned areas of each painting are outlined in white.

The paintings presented in this study come from tombs in and near the city center of modern day Paphos, Cyprus. Case Study 1 (*Painting of a woman*) is one of the rare examples of figurative painting within funerary contexts in Cyprus, recovered from Tomb 3510 (Figure 3a) [67]. This fragmented painting (∼0.7 m × 0.5 m) has been fully removed from the tomb (set into a plaster slab) and was analyzed in the storeroom of the Paphos Archaeological Museum. A small amount of discoloration and weathering on the painting’s decoration has occurred. In Paphos, only two other small, incomplete, fragmented faces exist in the entire excavated collection of funerary wall paintings. The beautiful, naturalistic execution of this figurative painting, as seen by the gradient of flesh tones through layering and mixing of various pigments (techniques described by ancient writers Pliny and Theophrastus [68,69]) can begin to fill the knowledge gap of artistic practices in Hellenistic and Roman Cyprus. Here, Case Study 1 will be used to exemplify the high-quality diffuse reflectance spectra of which the SRS hyperspectral system is capable of collecting, as well as accurate color information.

Case Study 2 is a wall painting located within Tomb 3882 in Paphos (Figure 2), which still remains in situ. This tomb was uncovered by accident during a modern-day construction project, resulting in its partial destruction, although a metallic structure was built to cover and protect the exposed site afterwards. The painting of interest (*Imitation marble*), located on the tympanum of the southeast arcosolium (∼1.6 m × 0.6 m) (Figure 3b), features a region of an imitation of marble revetment (a facing of masonry) delineated by incised lozenges. The practice of incorporating imitation marble into domestic and funerary wall paintings was prevalent in Roman wall painting, and studying marble decoration, particularly if it represents imported stones that were a luxury at the time, provides insight into the wealth, artistic culture, and socio-political status of the patrons [70]. While Tomb 3882 has suffered some destruction and environmental weathering, its discovery was relatively recent compared to others excavated in the city center and nearby necropolis and funerary complexes. Thus, the tomb’s paintings have suffered less over time in an exposed environment compared to other excavated tomb paintings. The painting *Imitation marble* is slightly degraded with localized surface discoloration and encrustation. Case Study 2 highlights the analytical workflow for image cubes collected by the SRS hyperspectral system to perform materials identification (pigments, degradation products, and conservation consolidants) and mapping.

In earlier analytical campaigns, spot analyses using fiber-optic reflectance spectroscopy (FORS) and portable XRF were performed, combined with photomicrography and forensic photography [71]. Those results will be used here for validation of and comparison with the SRS hyperspectral system’s performance.

## 2. Methods

### 2.1. Design of the SRS Hyperspectral System

The motivation for the design of the SRS hyperspectral system comes from the line-scanning AVRIS airborne hyperspectral imaging system, which collects high spectral resolution hyperspectral image cubes from 400 to 2500 nm using a whiskbroom scan mirror to feed the spectrometer [72,73]. In essence, the scan mirror allows the hyperspectral camera to collect each pixel that makes up the spatial lines sequentially. The motion of the airplane advances the hyperspectral camera forward to collect the next line. At the system design level, this approach, while limited in its area rate collection, avoids the need for complex and expensive imaging spectrometers and 2D focal plane arrays used in laboratory systems. In this research, we adopted a whiskbroom approach to raster scan the paintings’ surfaces; this has been done previously in heritage science investigations in both the VNIR and SWIR wavelength ranges to produce image cubes of historical paintings [74,75,76,77].

To implement the whiskbroom scan approach, the SRS hyperspectral system consists of three major components: (1) a field-deployable, battery-operable fiber-optic diffuse reflectance spectrometer; (2) a computer-controlled 2D mechanical *x*-*y* scanner; and (3) an incandescent battery-powered light source. This research employed a fiber-optic spectral radiometer, the ASD Fieldspec 3 (Malvern Panalytical, Malvern, UK). The Fieldspec 3 was selected because the instrument is highly portable and field-deployable; the analytical range spans the near-UV to SWIR (350 to 2500 nm), which provides access to materials with diagnostic signatures beyond those visible to NIR and eliminates the need for separate instruments; and the spectrometer is a popular instrument among geologists and archaeologists as well as cultural heritage scientists. The Fieldspec 3 has three internal diffraction gratings and three detectors, all of which operate during a single measurement. For data collection in the near-UV to NIR range, the instrument operates a silicon photo-diode array. The SWIR analytical range utilizes two InGaAs photo-diode detectors to collect from 1000 to 1830 nm and 1830 to 2500 nm. Reflected light is collected through a flexible fiber-optic cable. The fiber bundle in the probe consists of fifty-seven fibers. Nineteen of the fibers designated for VNIR measurements are 100 microns. The remaining thirty-eight are for SWIR measurements and measure 200 microns. The high spectral sampling (1.4 nm for 400 to 1000 nm and 2 nm for 1000 to 2500 nm, Table 1) allows material identification of minerals in relatively pure applications, as well as separation of constituents in pigment mixtures by analysis of diagnostic absorptions in the reflectance spectra.

The mechanical scanning system was conceptualized to allow the illumination source and the fiber-optic probe of the spectrometer to scan in 2D across the surface of wall paintings, in both horizontal and vertical configurations, for operation in challenging spaces such as in tombs, on scaffoldings, and in storage rooms. The 2D mechanical *x*-*y* scanner had several design requirements: (1) it must be lightweight, (2) supportable by a tripod, and (3) allow the scan plane to be adjusted (tilted) parallel to the wall painting. The illumination and optical collection head to be mounted on the scanner needed to provide uniform illumination during data collection. As the goal was to design a system that did not require extensive engineering, a commercial 2D scanner designed for computer-aided drawing was adapted according to these needs. To perform the 2D scan over the surface of a painting, the fiber-optic cable of the FieldSpec 3 was mounted onto the scanning arm of a motorized *x*-*y* drawing instrument (Axidraw V3, Evil Mad Scientist, Inc., Sunnyvale, CA, USA). The Axidraw V3 is a lightweight, structurally robust pen-plotter (∼2.2 kg) with a range of motion matching the dimensions of a US letter size paper. The scanning arm responsible for the pen operation runs along an aluminum extrusion with wheels. During data collection, the scanner is instructed to ‘draw’ a raster scan pattern using the Inkscape software, now holding an illumination source and the fiber optic instead of a pen. The scan speed for the raster was determined by the spot size, as the objective was to effectively collect one spectrum per ‘step’. The Axidraw V3 was customized to have the motorized *z*-stage of the pen holder and associated electrical cables removed and replaced with a fixed mounting plate to accommodate the fiber-optic probe. This reduced the size of the scanner, allowing it to be positioned closer to the surface of a painting. The maximum scan region of the Axidraw V3 is 216 × 292 mm, which would take approximately 90 min to scan, collecting a 4 mm × 4 mm sample size every 1 s (Table 1).

**Figure 4:**
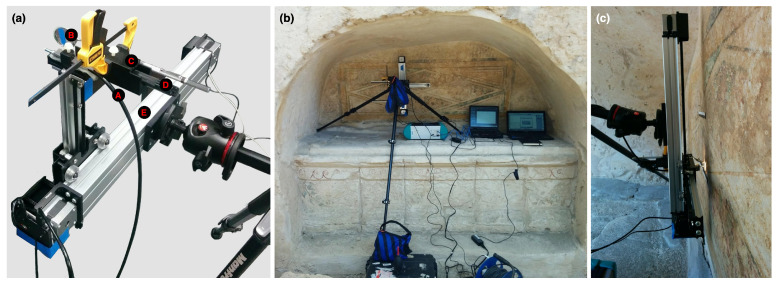
(**a**) The complete assembly of the scanning system: A—FO cable of the FieldSpec 3; B—mirror, clamped to mount—C; D—illumination source; E—scanner. (**b**) The SRS hyperspectral system set up to scan the painting *Imitation marble* in Tomb 3882. (**c**) Side profile of the scanning configuration.

A lightweight illumination source, a small battery-powered incandescent lamp (Mini Maglite, Ontario, CA, USA, ∼0.05 kg) emitting light from the near-UV to SWIR, was attached to the scanning arm. To record diffuse reflectance spectra, the fiber-optic probe and illumination source were mounted together onto the scanning arm using a custom-made 3D-printed holder, enabling the fiber optic to be kept perpendicular to the surface and the illumination to be projected on the surface at an angle of approximately 45° with respect to the fiber optic. This was achieved by keeping the Mini Maglite parallel to the fiber optic and by using a 31.75 mm diameter mirror (∼0.06 kg) to direct the light to the surface (Figure 4a). The SRS system, weighing approximately 2.5 kg in total, was mounted onto a tripod (MK190XPRO3, Manfrotto, Ramsey, NJ, USA) with a rotational ball head and adjustable center column, which can be oriented vertically or horizontally. This feature allowed the SRS hyperspectral system to be mounted in vertical and horizontal scanning configurations and to better match sloping surfaces of the paintings (Figure 4b,c).

Apparent diffuse reflectance spectra were collected using the acquisition software for the Fieldspec 3 instrument (RS^3^). The software converts the raw signal to apparent reflectance using a stored measurement off an ASD diffuse 98% reflectance standard, measured prior to the start of the image cube collection, along with a dark measurement. Each saved spectrum was an average of four collections each with an integration time of 136 ms. The spatial response function (3 to 4 mm in diameter) was set by the working distance from the painting’s surface, which was approximately 6–7 mm. The scanner was operated with a scan rate of 4 mm/s (Table 1), which matched the rate of the saved spectra (at 1 average spectrum per second) and the spatial response function. The irradiance at the painting from the Mini Maglite was ∼900 $\frac{W}{m^{2}}$ given the lamp was focused to an area of 3 cm in diameter and the output of the lamp is ∼9 lumens. The maximum temperature increase at the illuminated spot is less than 0.5 °C.

### 2.2. System Parameters

One of the most important parameters considered is the signal-to-noise ratio (SNR) of the spectra acquired. The SRS hyperspectral system noise level was evaluated by measuring the spectral radiance of the light source off a known reflectance standard and comparing it to the fiber optic spectrometer’s noise-equivalent spectral radiance (NESR) supplied by the vendor. The NESR is the spectral radiance at which the signal to noise is 1. The SNR for the SRS hyperspectral camera can be determined from the ratio of the measured spectral radiance, off a known target to the NESR. The spectral radiance was measured from a 98% diffuse white target using the ASD Fieldspec 3 spectroradiometer. Four measurements of the spectral radiance were made with the light source, the Mini Maglite, illuminating the diffuse white standard, and the average of four measurements was used to calculate the SNR. The SNR was determined at three wavelengths: 2100:1 at 700 nm, 1300:1 at 1400 nm, and 300:1 at 2100 nm, for a 98% diffuse white reflector (Table 1).

### 2.3. Data Analysis

Data from the two collections were first pre-processed by restructuring the thousands of point spectra acquired with the SRS hyperspectral system into an image cube using a Matlab script (MathWorks, Natick, MA, USA). Image cubes were then imported into ENVI (image processing and analysis software, L3Harris Geospatial, Boulder, CO, USA) to select out the paintings’ constituent materials and perform materials identification and mapping. Derivative calculations (both first and second derivative with respect to wavelength) were performed on the cubes in Matlab to emphasize diagnostic absorption features, inflection points, and peak locations, which are then compared to database spectra to identify minerals and dyes. This approach is often applied in remote sensing and diffuse reflectance spectra analysis to better separate admixtures, suppress background, and access more subtle features [78,79]. The SRS hyperspectral image cubes were therefore analyzed in several different spectral ranges in derivative space.

Spectral analysis tools in ENVI such as the Spectral Hourglass Wizard (SHW) and the Sequential Maximum Angle Convex Cone (SMACC) were used to find the spectral endmembers [80,81]. To determine the spatial distribution of these spectral endmembers within the image cube, the Spectral Angle Mapper (SAM) tool was used. The SAM algorithm calculates the angle between the vector describing the endmember spectrum and the vector associated with the spectrum at each spatial pixel in the image cube [82]. A spectrum with a smaller angle will have a higher degree of spectral similarity with that endmember. A tolerance angle is chosen to ensure pixels whose spectrums have an angle similar to or smaller than the angle will have the spectral features required to match the endmember spectra. The result of this process gives a map of the spatial pixels that have spectra that match well to the endmember. Next, in the classification step, an examination of the spectral features present in each endmember is done to assign them to a specific pigment or pigment mixtures. Principal component analysis (PCA) was also done to find eigenimages using the Minimum Noise Fraction (MNF) transform algorithm in ENVI [83]. Further detail on the data analysis procedure and tools mentioned above is described in [84].

## 3. Results

### 3.1. Case Study 1: Image Cube Reconstruction and Spectral Quality

**Figure 5:**
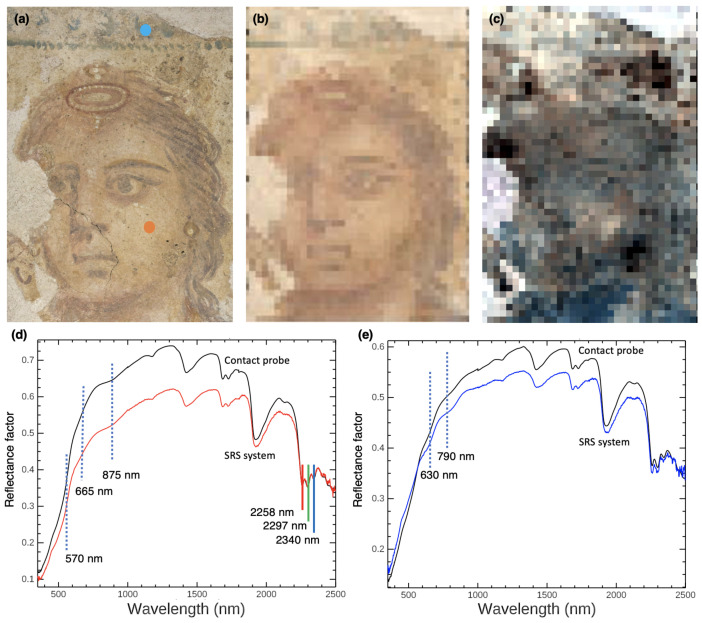
(**a**) Color photograph of the face in *Painting of a woman* (192 mm × 141 mm). (**b**) Reconstructed accurate color image from the reflectance image cube. (**c**) False-color SWIR image with blue, red, and green bands at 2258, 2297, and 2340 nm, respectively (these bands are marked in (**d**)). (**d**,**e**) Full-range SRS hyperspectral system spectra (350 to 2500 nm) from select pixels (locations marked in (**a**)) compared with FORS contact probe measurements at the same location. Characteristic absorptions and inflection points are marked by the dashed lines. (**d**) shows reflectance spectra from the flesh tone (red dot) in the figure’s proper left cheek (red—SRS system, black—contact probe), which have spectral features of red ochre in the VNIR spectral range (570, 665 and 875 nm). Spectra in (**e**) are reflectance spectra from the *aedicula* (blue dot) featuring Egyptian blue (blue—SRS system, black—contact probe), identified by absorptions at 630 and 790 nm.

An image cube was acquired of *Painting of a woman* from Tomb 3510. In this scene, the woman stands between two garlands in what seems to be an *aedicula*. The region of interest scanned is of the woman’s head and part of the *aedicula* (Figure 5a). The painting was positioned horizontally on the ground in the storerooms of the Paphos Archaeological Museum while the SRS hyperspectral system scanned the surface of the painting from above. The area scan was 192 × 141 mm. An accurate color image was calculated from the reflectance image cube using a D85 light source and the standard observers curves to calculate color coordinates (Figure 5b). The accurate color image shows the same features with similar hues to those in a color photograph acquired with a hand-held commercial camera, but with less color error. Clearly seen in the reconstructed image are the features of the face (eyes, nose, mouth, and hair), as well as the blue dot patterns above the face, believed to be part of the *aedicula* the woman was standing in [67], and the diadem in the hair. While the image looks pixelated, this is by design. The goal was not to oversample the spatial response function as is done in commercial cameras. While this would give a more pleasing image, it would also result in spectral mixing of adjacent pixels, which is not ideal for hyperspectral cameras. Rather, the SRS system was designed with one spatial sample (pixel) per spatial response function to ensure that little spectral mixing occurs between adjacent pixels, which also reduces the scan time five-fold.

The colorful pigments identified from the SRS spectral image cube of this painting include (but are not limited to) hematite-rich red ochre and goethite-rich yellow ochre, identified by their diagnostic Fe^3+^ crystal field transitions (to be discussed in more detail in the next case study) [85,86,87,88], and Egyptian blue (CaCuSi_4_O_10_), which has characteristic triple absorptions at 560, 630, and 790 nm [89,90,91]. A white pigment is also present in the diadem, which does not have characteristic features in the VNIR. Examples of the high-quality diffuse reflectance spectrum obtained at each pixel during the SRS system scan can be seen in Figure 5d,e, from spots collected on the woman’s proper left cheek and on the blue *aedicula*. The cheek shows features of red ochre, indicated by the inflection point and the NIR absorption at 875 nm. The blue pigment was assigned as Egyptian blue based on the 630 and 790 nm absorptions. These spectra were also compared to measurements collected at the same location by the Fieldspec 3’s contact probe (a handheld spot analyzer with an internal halogen bulb for contact analysis, with a near hemispherical solid angle collection compared to the SRS instrument). The contact probe’s larger spot size (10 mm) and higher irradiance (almost double compared to the SRS hyperspectral system configuration) means that the SNR of the contact probe will be higher by at least a factor of four to six. Ignoring the slight difference in overall intensities due to the different solid angles between the measurement configurations, the spectral features are the same between both collections for both spots. Despite the slightly lower SNR of the SRS hyperspectral system, the absorptions are clearly distinguishable and confidence can be placed in the measurements of this newly configured system.

In the SWIR, absorptions are present that correspond to different vibrational combination bands and overtones. Some of these features (1683, 1938, 2258 and 2298 nm) [92] can be ascribed to the CH, CH_2_, and CH_3_ stretching and bending modes of an organic consolidant, applied liberally over the surface of the painting. Others correspond to the presence of calcium carbonate (CaCO_3_) (∼2260, 2340 nm [93]), which is most likely responsible for the white pigment. Here, the possibility of overlap of some of the consolidant’s spectral features with CaCO_3_ is noted. While the deep-red portion of the SWIR shows higher noise than the visible and NIR ranges, these important absorptions in this range are still well-resolved such that the shifts and profiles of the absorptions can be noted. The relative intensity of these absorptions can also be used with this level of data quality to observe mixtures and changes in distribution across the painting. Figure 5c is a SWIR false color image, where R, G, and B channels are assigned to 2258, 2297, and 2340 nm, absorptions for the consolidant and CaCO_3_. For example, the blue pixels correspond to areas where the 2258 nm signal is weaker with respect to the 2340 nm carbonate feature.

The reduced intensity of the reflectance spectra in the image cube in regions of dark tones/lines, such as in the hair, eyes, and eyebrows, also underscores the presence of a black pigment, e.g., a manganese oxide-based colorant (pyrolusite, MnO_2_) or carbon black [94]. While the spatial sampling is not high, it is sufficient to show important spatial details of wall paintings with high spectral fidelity. In the next section, the advantage of the high spectral resolution for identification and mapping of materials using both the VNIR and SWIR spectral ranges is demonstrated.

### 3.2. Case Study 2: Spectral Endmember Extraction, Materials Identification and Chemical Mapping

**Figure 6:**
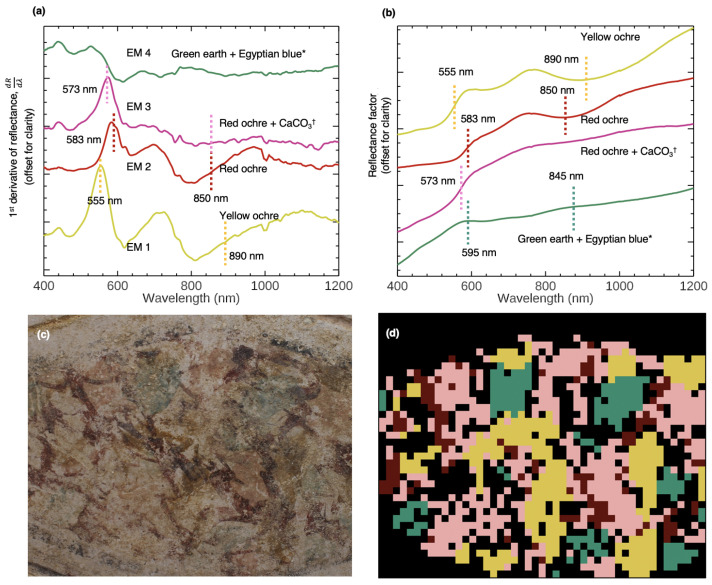
(**a**) The first derivative endmembers of Case Study 2, extracted from the SRS image cube of *Imitation marble*. (**b**) The reflectance equivalent endmembers of (**a**). * Egyptian blue was not identified by the SRS data. ^†^ CaCO_3_ was not identified in the VNIR analysis. (**c**) A color image of *Imitation marble* (266 mm × 150 mm). (**d**) The SAM map showing the distribution of the first derivative endmembers.

To better illustrate the capabilities of the system in unbiased identification and mapping of artists’ materials from the SRS image cubes collected on site, the painting *Imitation marble* in Tomb 3882, outlined in Figure 3b, was also analyzed. The scan area was 266 mm × 160 mm. Visual observations of the painting show that portions of the surface are weathered and contain encrustations. However, yellow, pink, red, and green paints used to create the marble decoration can be clearly seen. To find the endmembers of the pigments in the image cube, the analysis was focused on spectral features associated with electronic transitions within the 400–1200 nm range. Four unique first derivative endmembers were identified (Figure 6a), with their distribution visualized as a SAM map in Figure 6d. Single pixels in the first derivative cube that best match the extracted endmembers were used to identify pigments in the corresponding reflectance spectra (Figure 6b).

**Figure 7:**
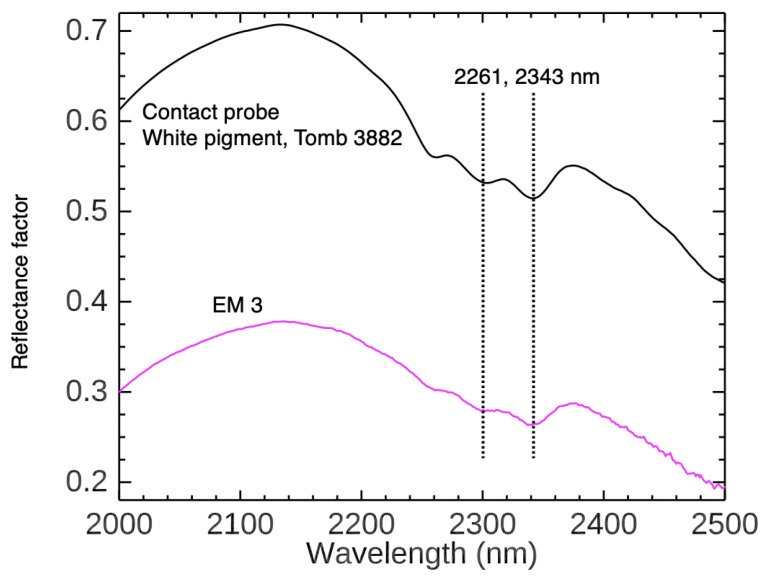
Comparison of the SWIR spectral ranges (2000 to 2500 nm) of EM 3, a mixture of red ochre and CaCO_3_, and a FORS measurement of a white pigment from a painted geometric design on another wall painting in Tomb 3882.

The endmember corresponding to the yellow paint—EM 1—was identified as goethite-rich yellow ochre by the first derivative peak at 550 nm (attributed to the Fe^3+^ crystal field transition for goethite) and its equivalent reflectance profile, featuring a shoulder at 450 nm, an absorption at 630 nm, a local maximum at 765 nm, and a NIR absorption at 892 nm [26,95]. These features match closely to the natural yellow ochre samples extracted from the Skouriotissa mine in Cyprus as measured by FORS. The endmembers representing the red and pink paint (EM 2 and 3, respectively) both contain hematite-rich red ochre. The first derivative and reflectance spectra of EM 3 show the characteristic spectral profile of red ochre (with an inflection at 575 nm due to the Fe^3+^ transition and a very subtle NIR absorption around 850 nm). The absorption features of red ochre appear more diluted and the VNIR profile has an overall increased reflectance compared to EM 2. This suggests the presence of a white pigment. To look for the inclusion of a possible white colorant, EM 2’s SWIR spectral range was analyzed (Figure 7). Spectral features at 2261 and 2343 nm corresponding to carbonate suggest that the artist mixed CaCO_3_ with red ochre to produce a pink hue. In the same tomb, another painting on the northwest arcosolium featuring a painted white geometric decoration was measured by FORS, and the white pigment was assigned as CaCO_3_ [71]. This result is not surprising, as CaCO_3_ was also identified as a white pigment in other ancient Cypriot wall paintings [56,64]. EM 2 from the imitation marble pattern appears to be mostly red ochre. The first derivative peak of the dark red endmember corresponding to the characteristic inflection is slightly shifted to 583 nm. The reduced relative intensities between the first derivative peaks from 500–800 nm and the overall reduced intensity of the reflectance profile suggest the presence of a dark material mixed into the red ochre or overlaid onto the surface. Color photographs of this region show thin layers of a black paint over select areas of the red ochre application. However, the 583 nm inflection, the local maximum at 753 nm, and the NIR absorption at 850 nm (though blue-shifted from reference hematite spectra) all indicate the presence of red ochre.

**Figure 8:**
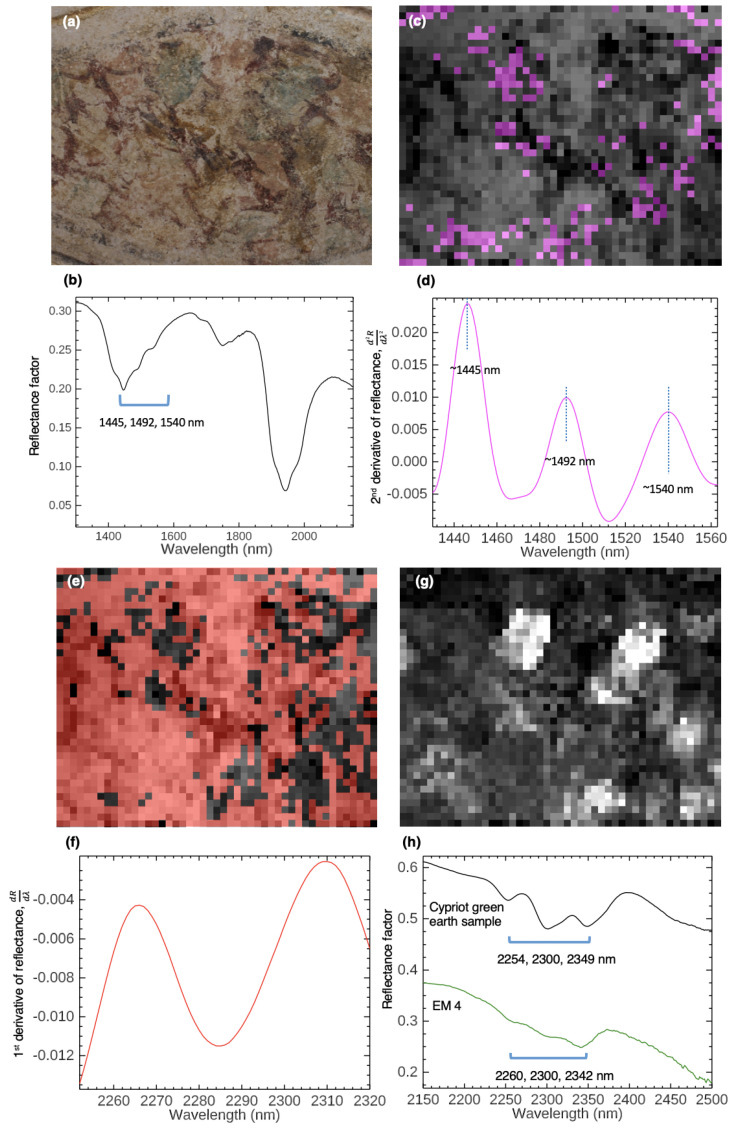
(**a**) Color image of the scan region of *Imitation marble*. (**b**) A SWIR spectrum from the reflectance image cube showing evidence of gypsum, identified by the triplet absorption from 1445 to 1540 nm and the water bands from 1940 to 1990 nm. (**c**) The SAM map of gypsum’s triplet signature (in magenta) using an endmember extracted from the second derivative-calculated cube subset from 1430 to 1563 nm (plotted in (**d**)), overlaid onto a greyscale image from the reflectance image cube. (**e**) The SAM map of PVA application over the surface of the painting (in red) using a first derivative endmember (plotted in (**f**)), overlaid onto a greyscale image from the reflectance image cube. (**g**) The MNF eigenimage from the PCA transform of the three broad spectral band images calculated from the SWIR portion of the image cube that shows the distribution of celadonite (in white). A reference SWIR spectrum from a local Cypriot green earth sample and the SWIR portion of EM 4 are in (**h**).

EM 4 corresponds to the blue-green paint, which has a green earth reflectance profile featuring a shoulder at 465 nm, a local reflectance maximum at 595 nm, absorption at 760 nm, and another local maximum at 845 nm. This spectrum matched closely to the spectral profile of a celadonite sample [96,97,98] from Cyprus. Celadonite-rich green earth was also previously identified by FORS in other ancient Cypriot wall paintings in earlier campaigns. This assignment, which makes sense in terms of geographical context, cannot rely on the VNIR alone for robust characterization. Celadonite and glauconite ((K,Na)(Fe^3+^,Al,Mg)_2_(Si,Al)_4_O_10_(OH)_2_), another natural green earth mineral (not native to Cyprus), have similar VNIR spectral profiles. However, celadonite has a triple absorption in the SWIR at ∼2257, 2302 and 2348 nm due to AlFe^3+^ and Mg OH combination bands, while glauconite has an absorption at 2317 and 2366 nm due to Fe^3+^ and Fe^2+^ OH combination bands [44]. The SWIR spectral region is necessary to reliably distinguish between celadonite and glauconite in paintings, as both were used as artist materials in ancient paintings. EM 4 has SWIR absorptions at 2260, 2300, and 2342 nm (Figure 8d), which confirms that this green earth pigment is celadonite-rich.

In a prior analytical campaign, the painting *Imitation marble* was imaged with a modified commercial color camera (the internal hot mirror removed) to search for any subtle presence of Egyptian blue, as no obvious blue paint was noted in the painting by visible observation. This was achieved by illuminating the painting with red light and filtering the camera with a NIR filter, as Egyptian blue has a diagnostic luminescence in the NIR at ∼900 to 1000 nm when excited by red light [99,100]. The analysis showed that Egyptian blue was present in the blue-green paint. Portable XRF analysis also showed significant levels of Cu K_α_ at 8.04 keV (along with the characteristic elements Fe, Mg, Si, and Al of green earth). Furthermore, photomicrography [71] also revealed coarse blue particles mixed with green particles. Together, these indicate that the paint is an admixture of Egyptian blue and celadonite (green earth); the ∼630 nm absorption in EM 4 can therefore be attributed to Egyptian blue.

One of the major causes of deterioration of the wall paintings is the formation of partially insoluble salts such as gypsum (CaSO_4_·2H_2_O). Owing to the presence of liquid moisture in the porous walls, mobilization of gypsum due to supersaturation and crystallization at the subsurface and surface of the paintings has caused severe damage and hard encrustations. Gypsum has a diagnostic triplet absorption feature in the SWIR at ∼1445, 1492, and 1540 nm (Figure 8b), corresponding to the hydroxyl (OH) first overtones and additional water combination bands at 1942 and 1988 nm. To better identify more subtle absorptions from 1480 to 1550 nm, especially in regions where the gypsum amount may be lower, a spectral subset of the region of 1430 to 1563 nm in the second derivative cube (turning the triplet feature’s absorptions into peak maxima) was analyzed. An endmember corresponding to gypsum featuring the triplet absorption was extracted using SMACC (Figure 8d). This endmember was mapped using the SAM tool, which shows the spatial distribution of gypsum, present either on the surface or still mobilizing through the sub-surface, or both (Figure 8c).

Absorption features in the spectra were also noted at 1683, 1938, 2258, and 2298 nm, which were assigned as polyvinyl acetate (PVA). The characterization was made based on comparison with reflectance spectra of PVA reference samples measured using FORS. PVA is a synthetic polymer resin used as a surface consolidant for wall paintings. This type of coating seemed to have been used in the conservation of different wall paintings, including both detached fragments kept in the Department of Antiquities (of Cyprus) storerooms and wall paintings remaining on site. A spectral subset of the SRS image cube from 2252 to 2323 nm was analyzed in the first derivative to identify an endmember with reflectance absorption features at ∼2258 and 2298 nm (Figure 8f). The SAM map shows a roughly uniform application of PVA over the surface of the wall painting (Figure 8e).

Principal component analysis (PCA) of three spectral band images created from the SWIR subset of the reflectance image cube was able to map a spectrally-identified pigment, celadonite green earth, that has weak characteristic SWIR absorptions in the dataset. The image cube’s discrete spectral bands from 1100 to 2400 nm were summed in ENVI to produce three wide spectral bands (1100 to 1400 nm, 1500 to 1800 nm, and 2100 to 2400 nm), which increased sensitivity to spectral features in these wavelength ranges [30,101]. A PCA transform was then performed on the three bands, giving a principal components image that represented the triplet SWIR infrared signature of celadonite (Figure 8g), as it is clear the white regions in the image correspond closely to the mapped regions of EM 4 in Figure 6d. This effectively demonstrates that the hyperspectral image cube can be used to create MSI datasets to utilize imaging processing techniques traditionally applied in MSI to map artist materials.

**Figure 9:**
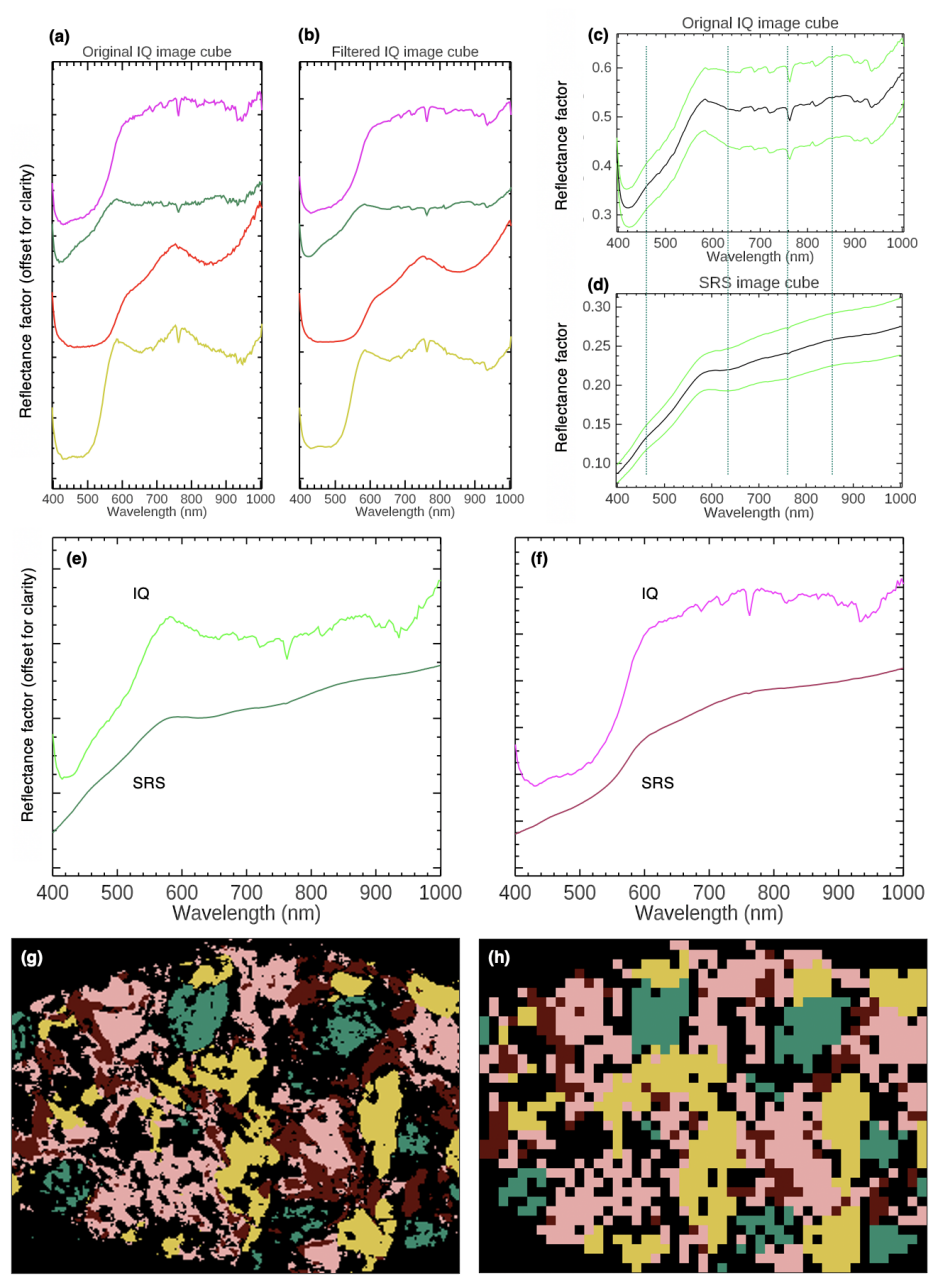
(**a**) The reflectance endmembers associated with those obtained from spectral analysis of the first derivative of the reflectance image cube of *Imitation marble* collected with the IQ hyperspectral camera. These endmembers are color-coded to match those obtained from analysis of the SRS image cube to allow comparison (see Figure 6b). (**b**) The four reflectance endmembers from (**a**) after filtering using an MNF transform on the cube. (**c**) An ROI was selected from the blue-green paint in the unfiltered IQ image cube from which the mean reflectance spectrum (the black curve) and the standard deviation (the green curves) were calculated. (**d**) An ROI was selected from the same region in the SRS image cube; the mean reflectance spectrum (the black curve) ± the standard deviation (the green curves) are plotted here for comparison. (**e**,**f**) compare the SRS image cube and IQ image cube reflectance endmembers for the blue-green and pink paint endmembers, respectively. (**g**) The IQ SAM map showing the distribution of the first derivative-cube extracted endmembers. (**h**) The SRS SAM map showing the distribution of the first derivative-extracted endmembers.

A comparison of the trade-off between high spatial/low spectral resolution and low spatial/high spectral resolution in the VNIR spectral region was also done against a small portable VNIR hyperspectral camera, the Specim IQ from Specim Corporation (400 to 1000 nm, 7 nm FWHM spectral resolution, 2.94 nm spectral sampling, 204 spectral channels, SNR for a 98% reflector is 280:1 at 700 nm) [102,103]. An image cube of *Imitation marble* was collected by the IQ camera. For this acquisition, a lamp was required to illuminate the area being imaged, and the spatial sampling was 1.5 mm, versus the 4 mm of the SRS. Analysis of the first derivative image cube, spatially aggregated by a factor of two in an attempt to improve the SNR two-fold, resulted in four endmembers. Figure 9a,b shows the equivalent reflectance spectral endmembers (best match in a single pixel) from the unaggregated, unfiltered-spectral and filtered-spectral (by an MNF transform) image cubes, respectively. In general, comparison of the spectra from the IQ with the SRS spectra shows the lower SNR in the IQ image cube as well as residual stray light from 400 to 425 nm in the spectrometer, which further impedes pigment identification.

A side-by-side comparison of the SAM maps in derivative space for the IQ and SRS image cubes show similar mapping of the endmembers (Figure 9g,h). However, only two of the endmembers obtained from analysis of the IQ image cube closely match the reflectance endmembers from the SRS image cube–EM 1 (yellow ochre) and EM 2 (red ochre)–while the two seeming to correspond to the SRS system’s EM 3 (Egyptian blue and green earth mixture) and EM 4 (red ochre and CaCO_3_ mixture) were quite different. It was not possible to identify characteristic spectral features of red ochre in the IQ’s pink endmember (similar to EM 3 from the SRS image cube analysis), such as the peak maximum at ∼760 nm and NIR absorption at ∼850 nm, which are necessary to couple with the wavelength position of the inflection peak in the first derivative for identification. Even averaging the IQ data does not result in significant improvement of the absorption features for assignment. A region of interest (ROI) in the blue-green paint was selected where the best match for the reflectance endmember appeared. A similar ROI was extracted from the SRS image cube for comparison. The mean and standard deviation of the spectra in the ROI were then calculated (Figure 9c,d). The noise level in the IQ image cube spectra was so high that it only appeared as though one large absorption was present in the NIR, and the characteristic reflectance features of Cypriot green earth at ∼760 and 845 nm in the NIR could not be distinguished, nor the 630 nm absorption of Egyptian blue. Though the spectral content in the IQ image cube was sufficient to separate the four pigments, the quality of the spectra was not sufficient to confidently characterize most of the endmembers in this less than challenging test.

## 4. Discussion

### 4.1. System Design Evaluation

The SRS hyperspectral system exhibits strong performance in signal to noise for the visible and NIR wavelength ranges and moderate performance in the SWIR. Case Study 2 of *Imitation marble* shows how access to vibrational spectral features in the SWIR can provide additional insight into a painting’s materials and condition. Many conservation and cultural heritage science laboratories have FORS instruments, but not imaging spectrometry capabilities, due to the high cost of the instruments. This system shows that a standard FORS instrument can easily be adapted into a scanning configuration to produce high spectral resolution image cubes of paintings and other 2D decorated works of art to achieve the same data output as imaging spectrometry systems. The cost of the scanner, tripod, illumination source, and mounting accessories summed to approximately $900. The ASD Fieldspec 3, already in the possession of the UCLA group, cost approximately $70k. No commercial imaging system exists that covers this spectral range with this spectral resolution. The systems closest in performance covering the 400 to 2500 nm range operate at two to three times the spectral sampling and are four times the cost of the ASD Fieldspec 3.

The main challenges experienced by the current configuration of the SRS hyperspectral system were highly uneven surfaces and a limited field of regard (as dictated by the Axidraw *x*-*y* scanner). However, the system is highly configurable and can be modified to make advantageous improvements. A different scanner with a larger range of translation that can adjust its position in the *z*-dimension would allow the fiber optic probe to run across uneven surfaces at a set working distance. Adding a lens to the system could also bring the spot size down to 1 mm, and the Axidraw scanner can raster at 1 mm/s, increasing the spatial resolution of the image cube. At this scan rate, data collection time is comparable to that of macroscale XRF scanning systems (e.g., 0.25 mm/pixel collecting at 40 ms/pixel). However, the scan time can also be improved (e.g., reduced) by integrating a different spectrometer that operates at a spectrum acquisition rate. Furthermore, image cubes of paintings larger than the available scan range can be produced by mosaicking individual scans [104]. This has been done for fine art paintings to create image cubes of complete large-scale artworks without compromising spatial resolution [48]. The application of mosaicking is particularly valuable for confined areas, such as tombs, to produce comprehensive image cubes of their wall paintings.

**Figure 10:**
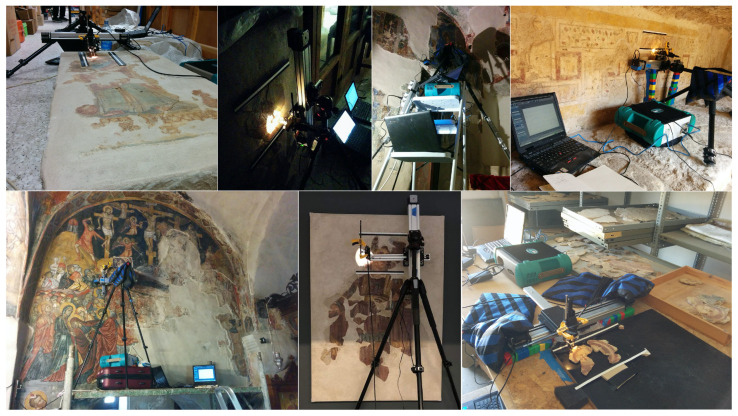
The SRS hyperspectral system was applied in various locations in/near Paphos, Cyprus, including ancient tombs, religious spaces, museum galleries and laboratories, and field storerooms.

The SRS hyperspectral system examples shown here use a generator to power the scanner and Fieldspec 3. However, both components can be battery-operated. On the other hand, laboratory-based imaging spectrometry systems, or even the portable IQ VNIR hyperspectral camera, require the use of lamps for broad illumination, which necessitates a power supply. For sufficient and uniform illumination, two to four lamps are often needed, which makes imaging spectrometry less amenable to field analyses. The portability of the spectroradiometer and the *x*-*y* scanner made it possible to build an integrated system that was field-deployable and versatile in application. The SRS hyperspectral system has been used in various non-laboratory settings to produce image cubes of both ancient and Byzantine Cypriot wall paintings (Figure 10).

### 4.2. Materials Identification and Mapping

The measured signal of the system is proportional to (spatial sampling) × (spectral band width) × (radiance in the spectral band) × (exposure time) for a fixed solid angle. Spatial sampling had to be sacrificed in the system design to increase exposure time, as the collection time scales as the square of the sampling for a fixed SNR and illumination. It was demonstrated that the spatial sampling of the SRS system was sufficient to find the spectral endmembers representative of the painting. Analysis of the IQ image cube featuring high spatial resolution showed that sacrificing spatial resolution in the SRS system design did not result in overlooked spectral endmembers.

Imaging spectrometry systems with 7–10 nm resolution, such as the IQ hyperspectral camera, might be able to distinguish relatively pure pigment applications, but pigment mixtures, such as the red and yellow ochres found in flesh tones combined with other pigments that comprised much of ancient artists’ palettes [105,106], are more difficult to resolve. Additionally, distinguishing between materials with distinct, shifted absorption features such as red lakes in the 500–600 nm range [107] or red semiconductor pigments with characteristic absorption edges and full width-half maxes [35] also requires higher spectral specificity. This is also relevant for identification of minute diagnostic absorptions, such as the 433–437 nm absorption of jarosite ((K,Na)Fe_3_^3+^(OH)_6_(SO_4_)_2_), where a shift at these wavelengths distinguishes between K or Na dominant jarosite [45,108]. These materials are all prominent in ancient painting, making the high spectral resolution a necessity.

Chemical maps of a painting’s pigments, weathering products, and previously-applied conservation materials can serve as base maps for ongoing environmental monitoring. Successive captures over time with updates to the chemical mapping of these signatures can provide continuous insight into the painting’s stability. However, the capabilities of this system have not been fully exploited in this study because the materials present in these tomb paintings are few compared to the diversity of artist materials, alteration/degradation products, and conservation materials that are found on wall paintings in other geographical contexts and time periods. As previously mentioned, distinguishing between the use of glauconite and celadonite is critical to the characterization of green earth pigments that have been used in artworks since antiquity [109,110]. Line or preparatory drawings in carbon black can also be detected and mapped, as many pigments are transparent into the SWIR, allowing carbon black to be visualized at longer wavelengths [111]. Other organic materials besides PVA such as wax have often been used as consolidants in the conservation of wall paintings. Wax has characteristic absorptions at 1730, 1763, 2311, and 2352 nm from CH_2_ vibrational overtones, and fundamental modes that can be distinguished from other lipidic painting materials such as oil binders and rabbit skin glue with high resolution spectra [36,112]. It may also be possible, if present in appreciable amounts, to identify the types of clays present in paints composed of mined pigments powdered from extracted rocks (e.g., kaolinite, illite, montmorillite, etc.) [113].

Although not implemented in this study, further data processing can also be applied benefitting from access to high resolution SWIR data, such as linear spectral unmixing and semi-quantitative absorption depth analyses, as well as other chemometric approaches [114,115,116,117,118]. The ability to record high spectral resolution image cubes in the SWIR opens possibilities for in-depth materials analysis and mapping for on-site wall paintings, which the SRS hyperspectral system has begun to demonstrate with these archaeological case studies.

## 5. Conclusions

This research has presented the design and implementation of a moderate cost, highly portable scanning reflectance spectrometry system able to produce image cubes with high spectral resolution from the near-UV to SWIR. This instrument afforded access to spatially-resolved important spectral signatures in a painting that reflect its manufacture, condition, and conservation treatment. When applied for the analysis of wall paintings from archaeological tombs in Paphos, Cyprus, this system achieved the identification and mapping of constituent pigments, weathering products, and conservation materials. It has been demonstrated that as a standalone instrument, the reduced spatial resolution compared to traditional imaging spectrometry systems in this simplified design did not present a significant drawback in image reconstruction, materials identification, and mapping outcomes. Furthermore, ancient paintings were able to be scanned in high spectral detail, producing extensive documentation of their material makeup and ongoing transformations. The high sensitivity of the detector and statistical analyses allows researchers to re-visualize deteriorated regions of the painting, versus relying on biased visible observation prevalently used in archaeological painting reconstructions. Results from this study provide a strong argument for future development and optimization of field-deployable reflectance spectrometry scanning systems for improved on-site analyses of wall paintings and other immovable heritage.

## Figures and Tables

**Table 1:** SRS hyperspectral system technical specifications.

Spectral range	350 to 2500 nm
Spectral sampling	1.4 nm (VNIR)—2 nm (SWIR)
Spectral resolution (FWHM)	3 nm (VNIR)—10 nm (SWIR)
Signal to noise (for 98% reflector)	2100:1 (700 nm), 1300:1 (1400 nm), 300:1 (2100 nm)
Spatial sampling	4 mm^2^/pixel
Spatial response (with no collection lens)	4 mm (dia.)
Scan region	Max. 216 mm × 292 mm
Maximum image cube size (using current scanner)	54 × 70 pixels spatial pixels by 2151 spectral channels
Scan rate	4 mm/s

## Data Availability

Data available from authors upon reasonable request.
